# A Rare Case of Acute Renal Failure Secondary to Rhabdomyolysis Probably Induced by Donepezil

**DOI:** 10.1155/2014/214359

**Published:** 2014-04-27

**Authors:** Osman Zikrullah Sahin, Teslime Ayaz, Suleyman Yuce, Fatih Sumer, Serap Baydur Sahin

**Affiliations:** ^1^Department of Nephrology, Recep Tayyip Erdogan University Medical School, 53100 Rize, Turkey; ^2^Department of Internal Medicine, Recep Tayyip Erdogan University Medical School, 53100 Rize, Turkey; ^3^Department of Endocrinology and Metabolic Disease, Recep Tayyip Erdogan University Medical School, 53100 Rize, Turkey

## Abstract

*Introduction*. Acute renal failure (ARF) develops in 33% of the patients with rhabdomyolysis. The main etiologic factors are alcoholism, trauma, exercise overexertion, and drugs. In this report we present a rare case of ARF secondary to probably donepezil-induced rhabdomyolysis. *Case Presentation*. An 84-year-old male patient was admitted to the emergency department with a complaint of generalized weakness and reduced consciousness for two days. He had a history of Alzheimer's disease for one year and he had taken donepezil 5 mg daily for two months. The patient's physical examination revealed apathy, loss of cooperation, and decreased muscle strength. Laboratory studies revealed the following: urea: 128 mg/dL; Creatinine 6.06 mg/dL; creatine kinase: 3613 mg/dL. Donepezil was discontinued and the patient's renal function tests improved gradually. *Conclusion*. Rhabdomyolysis-induced acute renal failure may develop secondary to donepezil therapy.

## 1. Introduction

Rhabdomyolysis is defined as a clinical condition in which damaged skeletal muscle breaks down rapidly, releasing toxic substances such as creatine kinase (CK) and myoglobin into the bloodstream [1]. The severity of symptoms depends upon the extent of muscle damage and development of renal failure [2, 3]. It may be asymptomatic or have symptoms including muscle pains, vomiting, and confusion. Some patients may develop severe hypovolemia, shock, arrhythmia, and acute renal failure (ARF) [4, 5].

ARF develops in 33% of the patients with rhabdomyolysis [6]. The main etiologic factors are alcoholism, trauma, exercise overexertion, sunstroke, heat intolerance, hypophosphatemia, convulsions, infections, ischemia, and drug use or overdose [3, 7, 8]. Drugs reported to induce rhabdomyolysis include cocaine, amphetamines, statins, fenofibrate, heroin, corticosteroids, and colchicine [3, 7, 8].

In this report we present a rare case of ARF secondary to rhabdomyolysis probably induced by donepezil.

## 2. Case Report

An 84-year-old male patient was admitted to the emergency department with a complaint of generalized weakness and reduced consciousness for two days. He had a history of Alzheimer's disease for one year and he had taken donepezil 5 mg daily for two months. He had no other diseases and he had not taken any other medications. He had no history of trauma, convulsion, previous fall, or alcohol intake.

The patient's physical examination revealed apathy, loss of cooperation, and decreased muscle strength. His temperature was 36.8°C, blood pressure 140/90 mm/Hg, and pulse rate 88 bpm. He had bilateral moderate pretibial edema.

Laboratory studies revealed the following: urea: 128 mg/dL; creatinine: 6.06 mg/dL; aspartate aminotransferase: 93 U/L; CK: 3613; calcium: 8.1 mg/dL; phosphorous: 4.9 mg/dL; sodium: 149 mmol/L; potassium: 4,3 mmol/L; albumin: 3.7 g/dL; lactate dehydrogenase: 349 U/L; hemoglobin: 14.2 g/dL; fT3: 3.5 (N: 1.71–3.71 pg/mL); fT4: 1.35 (N: 0.7–1.48 ng/dL); TSH: 2.04 (N: 0.35–4.94 uIU/mL). Urinary dipstick analysis was 1+ positive for protein and 3+ positive in the Haem test. Urinary sediment showed a few red blood cells and 2-3 leukocytes per high-power field. Arterial blood gases analysis was PH: 7.44, PCO_2_: 23 mmHg, PO_2_: 151 mmHg, SO_2_: 99.5%, and HCO_3_: 19 mmol/L.

The patient's renal function tests were performed by other health centers before two months and they were completely normal. His renal ultrasound evaluation was normal. The patient was evaluated by a neurologist and there was no neurologic pathology other than Alzheimer's disease. Echocardiography was performed and ejection fraction was 60%, left ventricle was concentric hypertrophic, and a minimal pericardial effusion was reported. The patient was admitted to the nephrology ward with a diagnosis of ARF. Donepezil was discontinued. There was no indication for emergent hemodialysis. Intravenous hydration therapy was given. The patient's renal function tests improved gradually and were normal after 12 days of the treatment. He was discharged with complete recovery.

## 3. Discussion

We have reported a rare case of rhabdomyolysis associated with donepezil treatment that had progressed to ARF. In literature there was only one case report of rhabdomyolysis related to donepezil treatment in a 76-year-old man with type 2 diabetes mellitus and pulmonary emphysema who had been diagnosed with Alzheimer's and recovered shortly after discontinuing the drug [9]. The patient in our case had no history of trauma, convulsion, exercise, or alcohol intake and he had not taken any other medications such as statin or colchicine that may induce rhabdomyolysis.

Donepezil is a centrally acting reversible acetylcholinesterase inhibitor (ChE) [10]. It is used in palliative treatment of mild to moderate Alzheimer's disease [11]. The safety of donepezil has been demonstrated in many studies ranging from 12-week to 5-year duration [12–15]. Renal function is known to be decreased with increasing age [16]. Donepezil has been found also to be safely administered to patients with moderate to severely impaired renal function [17].

In our case ARF was a result of rhabdomyolysis probably induced by donepezil and not a direct nephrotoxic effect of the drug. It is not known how donepezil can cause rhabdomyolysis. Further studies may be needed to clarify this effect. The routine control measurement of CK may be suggested after administration of donepezil or other drugs that may have rhabdomyolysis effect in elderly patients. Clinicians should be alert for rhabdomyolysis in patients with generalized body weakness and muscle pain. Early management in these patients is vital in protecting the renal functions and preventing morbidity and mortality.

In conclusion, rhabdomyolysis may be associated with many medications. So drugs should be given carefully to the patients particularly the aged or those with chronic diseases and patients should be warned of possible side effects.
